# Classical Seminoma With Secondary Undescended Testis in an Elderly Patient: An Incidental Finding With a Penile Fracture

**DOI:** 10.7759/cureus.31924

**Published:** 2022-11-26

**Authors:** Belal N Sabbah, Abdulaziz Alathel, Mohammad A Alghafees, Ahmad N Sabbah, Omar Alfraidi, Naif Althonayan, Mohammed Shareef, Abdullah Alkhayal, Saad Abumelha

**Affiliations:** 1 College of Medicine, Alfaisal University, Riyadh, SAU; 2 Urology, King Abdulaziz Medical City Riyadh, Riyadh, SAU; 3 College of Medicine, King Saud Bin Abdulaziz University for Health Sciences, Riyadh, SAU; 4 Medicine, King Abdullah International Medical Research Center, Riyadh, SAU; 5 Urology, National Guard Hospital, Riyadh, SAU; 6 Urology, King Fahad Hospital, Madinah, SAU; 7 College of Medicine, King Saud bin Abdulaziz University for Health Sciences, Riyadh, SAU; 8 Department of Surgery, Ministry of National Guard - Health Affairs, Riyadh, SAU

**Keywords:** urology, inguinal hernia, penile fracture, seminoma, cryptorchidism

## Abstract

Cryptorchidism is a common condition among children; however, it is rare in adults and is associated with an increased risk of malignancy. The development of secondary undescended testes is recognized as a complication following inguinal surgeries such as hernia repair and orchidopexy. Herein, we describe the case of a 64-year-old male with a known past surgical history of right indirect inguinal hernia repair complaining of penile swelling. The patient was diagnosed with a penile fracture, and a genital examination further revealed a right undescended testis. The patient underwent penile fracture repair and right orchiectomy. Histopathology examinations showed classic seminoma. These findings show that the position of each testis should always be documented before, during, and after inguinal hernia repair due to the increased risk of undescended testis. Histopathological confirmation is necessary for such patients so that malignancy can be identified in its early stages and cured subsequently.

## Introduction

Cryptorchidism (undescended testis) is the failure of the testis to descend to its normal position in the base of the scrotum. It is a common finding in the pediatric population, affecting 2% to 4% of male infants [1]. However, cryptorchidism is an uncommon finding in the adult age group. Its prevalence reaches about 1% by the age of 1 year, and few cases have been diagnosed and treated in patients older than 1 year [2]. Furthermore, it is rare to find a patient diagnosed after the age of 60 years [3]. Cryptorchidism is an important risk factor for testicular malignancy, particularly testicular germ cell cancer [4]. Classic seminoma accounts for 85% of seminomas. This cancer occurs commonly between 40 and 50 years of age [3,4]. Herein, we describe the case of a 64-year-old male with a known past surgical history of right indirect inguinal hernia repair complaining of penile swelling, who ultimately was diagnosed with a seminoma.

## Case presentation

A 64-year-old male presented to the emergency room with a complaint of penile swelling for four days. He reported hearing a click sound while having regular sexual intercourse. The patient waited for the swelling to resolve spontaneously; however, it progressed over time. His past medical history was significant for ischemic heart disease.

Surgical history showed that the patient had a right indirect inguinal hernia repaired 30 years prior.

**Figure 1:**
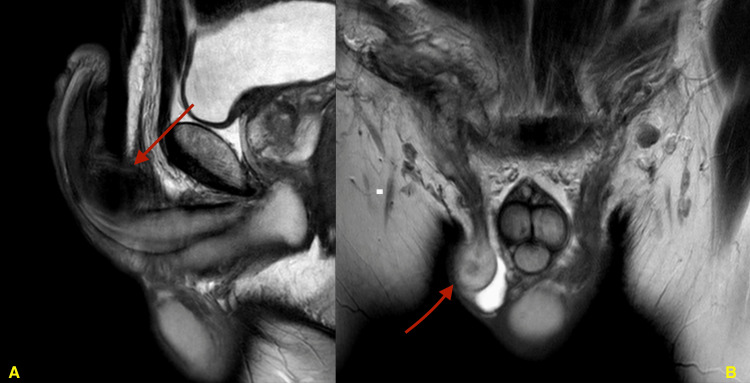
(A) Sagittal view of the MRI showing a penile fracture. (B) Coronal view of the MRI showing a right undescended testicle. MRI, magnetic resonance imaging

On examination, the abdomen was soft, lax, and non-tender. The patient's genital examination showed generalized penile swelling with ecchymosis extending to the umbilicus, scrotum, and perineum. Scrotal examination revealed a well-developed scrotum, left normal testis in shape, size, and consistency, and empty right hemiscrotum. The patient's baseline lab investigations were in the normal range. The medical team ordered an MRI of the abdomen and pelvis. The MRI revealed a penile root tunica albuginea defect at the lateral aspect of left corpora cavernosa, and small-sized right testis located at the neck of the scrotum with heterogeneous enhancement (Figure 1).

The medical team suspected testicular cancer based on the patient's persistent cryptorchid right testis. A CT of the chest was ordered to look for distant metastasis, but it was unremarkable. A surgery combining primary repair of the penile fracture and right radical orchiectomy was planned. The surgery was uneventful, and the right testis was sent for a histopathology examination. Surprisingly, the histopathology report showed the classic type of seminoma, which is extremely rare in this age group.

The medical team diagnosed the case as secondary undescended testis. As per the patient’s history, he had right undescended testis even before the surgery was performed for an indirect inguinal hernia. The undescended testis was identified and simultaneously repositioned during the surgery. However, the testis lost its normal position again after the hernia repair. This could be due to either primary orchidopexy failure or an iatrogenic complication of hernia repair surgery, thus resulting in secondary undescended testis.

## Discussion

Cryptorchidism is a very common anomaly of the male genitalia. It is more common in infants, but rare in adults. Around 10% of all cases of testicular germ cell tumors (TGCT) occur in men with a history of cryptorchidism [5]. Although the incidence of TGCT is higher in young adults, they are rarely present in senior adults. The prevalence of TGCTs in patients above 65 years of age is less than 4% [3]. The most common type of TGCT in cryptorchidism is seminoma [6].

Cryptorchid testes can be found coexisting with inguinal hernias, as seen in our case where the patient had right cryptorchidism and later on developed a right indirect inguinal hernia. In one case reported by Zuiki et al., the authors described an inguinal hernia with cryptorchidism in an elderly patient [7]. Secondary undescended testes is a phenomenon that may occur after an inguinal hernia repair or as a complication of orchidopexy [8]. There is sufficient evidence in the literature regarding the failed outcomes of orchidopexy, resulting in testicular retraction presenting as undescended testes [7,9]. The development of cryptorchidism after the inguinal hernia repair surgery in our patient may be explained either by mechanical tethering of the testis in scar tissue or failed orchidopexy.

The diagnosis of asymptomatic classical seminoma in elderly patients is typically an incidental finding. Most articles reviewed in the literature showed a declining incidence of testicular GCTs beyond the age of 40 [10,11]. There are few published case reports and case series in which authors reported cases of classic testicular seminoma in men aged 50 years or over [12]. Furthermore, clinical characteristics of seminomas in the elderly differ per patient. The most common presenting symptom in older adults, as well as young adults, is a palpable and painless scrotal mass that can be associated with discomfort [13]. This case is unique in the way that the patient was asymptomatic until he had the penile fracture.

The treatment of classical seminoma may be divided surgically and medically. Surgically, a radical inguinal orchiectomy is performed. However, sperm cryopreservation should be offered prior to initiating therapy. From a medical standpoint, adjuvant radiotherapy and chemotherapy are given based on the clinical staging group and the prognosis [14]. Active surveillance is recommended in stage I seminoma, while in stages II and III chemotherapy and radiotherapy are indicated [14].

## Conclusions

Classical seminoma in a patient with secondary undescended testis is seldom to be expected in a 64-year-old man. This encourages meticulous history taking and clinical examination for all patients by documenting the position of each testis before, during, and after inguinal hernia repair. If undescended testes are found, even if the patient is asymptomatic, a radical orchidectomy with histopathological examination should be performed to rule out any malignancy because of the increased risk of malignancy in persistent cryptorchid testes.
